# The Genetic and Epigenetic Architecture of Keratoconus: Emerging Pathways and Clinical Implications

**DOI:** 10.3390/genes17010066

**Published:** 2026-01-06

**Authors:** Francesco Cappellani, Matteo Capobianco, Federico Visalli, Cosimo Mazzotta, Fabiana D’Esposito, Daniele Tognetto, Caterina Gagliano, Marco Zeppieri

**Affiliations:** 1 Department of Medicine and Surgery, University of Enna “Kore”, Piazza dell’Università, 94100 Enna, Italy; francesco.cappellani@unikore.it (F.C.); f.desposito@imperial.ac.uk (F.D.); caterina.gagliano@unikore.it (C.G.); 2 Eye Center, G.B. Morgagni-DSV, 95100 Catania, Italy; 3 Department of Ophthalmology, University of Catania, 95123 Catania, Italyfederico.visalli13@gmail.com (F.V.); 4 Department of Medicine, Surgery and Health Sciences, University of Trieste, 34127 Trieste, Italy; 5 Department of Ophthalmology, University Hospital of Udine, 33100 Udine, Italy

**Keywords:** keratoconus, genetics, epigenetics, genome-wide association studies, microRNAs, DNA methylation, extracellular matrix, personalized medicine

## Abstract

Background: Keratoconus (KC) is a progressive corneal ectasia and a leading cause of corneal transplantation in young adults. Once regarded as a biomechanical disorder, KC is now recognized as a complex disease driven by genetic predisposition, epigenetic modulation, and environmental triggers. Advances in genomics and transcriptomics have begun to elucidate the molecular mechanisms underlying corneal thinning and ectasia. Objectives: This review synthesizes two decades of evidence on the genetic and epigenetic architecture of keratoconus, highlights key molecular pathways implicated by these findings, and discusses translational implications for early diagnosis, risk prediction, and novel therapeutic strategies. Methods: A narrative review was conducted of peer-reviewed human, animal, and in vitro studies published from 2000 to 2025, with emphasis on genome-wide association studies (GWAS), sequencing data, methylation profiling, and non-coding RNA analyses. Findings were integrated with functional studies linking genetic variation to molecular and biomechanical phenotypes. Results: Genetic studies consistently implicate loci such as *ZNF469*, *COL5A1*, *LOX*, *HGF*, *FOXO1*, and *WNT10A*, alongside rare variants in Mendelian syndromes (e.g., brittle cornea syndrome, Ehlers–Danlos spectrum). Epigenetic research demonstrates altered DNA methylation, dysregulated microRNAs (e.g., MIR184, miR-143, miR-182), and aberrant lncRNA networks influencing extracellular matrix remodeling, collagen cross-linking, oxidative stress, and inflammatory signaling. Gene–environment interactions, particularly with eye rubbing and atopy, further shape disease expression. Translational progress includes polygenic risk scores, tear-based biomarkers, and early preclinical studies using RNA-based approaches (including siRNA and antisense oligonucleotides targeting matrix-degrading and profibrotic pathways) and proof-of-concept gene-editing strategies demonstrated in corneal cell and ex vivo models. **Conclusions**: Keratoconus arises from the convergence of inherited genomic risk, epigenetic dysregulation, and environmental stressors. Integrating multi-omic insights into clinical practice holds promise for earlier detection, precision risk stratification, and development of targeted therapies that move beyond biomechanical stabilization to disease modification.

## 1. Introduction

Keratoconus (KC) is a progressive, degenerative corneal disorder characterized by stromal thinning and conical protrusion of the cornea. Clinically, it leads to irregular astigmatism, myopic shifts, corneal scarring, and vision loss in advanced cases. Traditionally considered a non-inflammatory ectasia, KC is now understood as a multifactorial disease involving a complex interplay of genetic predisposition, environmental/behavioral triggers, and biochemical factors [1].

A familial inheritance pattern, higher concordance in monozygotic twins, and ethnic disparities in prevalence all point to a strong genetic component [2,3]. At the same time, external factors such as chronic eye rubbing, atopy (allergic disease), nutritional factors, contact lens wear, and UV exposure are known risk modifiers that can precipitate or exacerbate keratoconus in susceptible individuals [4].

Over the past 20 years, tremendous progress has been made in deciphering the genetic and epigenetic basis of keratoconus. Genome-wide association studies (GWAS) have identified numerous common single-nucleotide polymorphisms (SNPs) associated with KC across diverse populations [5]. While candidate gene approaches and family-based linkage studies have uncovered both common and rare variants in genes related to corneal structure and repair [6]. In parallel, emerging research in epigenetics—including DNA methylation profiling, histone modification analysis, and non-coding RNA expression—has illuminated additional layers of regulation that contribute to the KC phenotype. These genomic and epigenomic discoveries converge on disruptions in key molecular pathways, such as extracellular matrix (ECM) organization, collagen cross-linking, oxidative stress responses, and inflammatory signaling, which together drive the corneal thinning and biomechanical weakening characteristic of keratoconus [7,8].

In this narrative review, we comprehensively examine peer-reviewed studies from the past two decades that investigate the genetic and epigenetic underpinnings of keratoconus, drawing on evidence from human clinical genetics, animal models, and in vitro experiments. We highlight the major genetic risk loci and genes (both common and rare) implicated in KC discuss syndromic overlaps that have provided etiologic clues, and summarize epigenetic mechanisms (DNA methylation changes, histone marks, microRNAs, long non-coding RNAs) that modulate corneal gene expression. We also review gene–environment interactions such as eye rubbing and atopy, which provide insight into how genetic susceptibility and external factors jointly influence disease onset and progression. Throughout, we note functional studies that have explored how these genetic/epigenetic factors alter molecular pathways—for example, how dysregulation of ECM components or antioxidant enzymes can lead to corneal instability. Finally, we consider the clinical implications of these findings, including prospects for earlier diagnosis via genetic/epigenetic biomarkers, personalized risk prediction (e.g., polygenic risk scores), and emerging therapeutic approaches that target the molecular causes of keratoconus (such as RNA-based treatments or future gene editing strategies).

## 2. Materials and Methods

A narrative literature review was conducted to synthesize current evidence on the genetic, epigenetic, and molecular basis of keratoconus. Peer-reviewed studies published in English between January 2000 and October 2025 were identified through comprehensive searches of PubMed, Scopus, and Web of Science databases. The following key terms and combinations were used: “keratoconus,” “genetics,” “GWAS,” “sequencing,” “mutation,” “epigenetics,” “DNA methylation,” “histone modification,” “microRNA,” “lncRNA,” “pathogenesis,” and “corneal biomechanics.” Original research articles, meta-analyses, and systematic reviews involving human subjects, animal models, or in vitro systems were included if they provided molecular or functional insights relevant to keratoconus pathophysiology. Inclusion criteria comprised peer-reviewed original research articles, meta-analyses, and authoritative reviews involving human subjects, animal models, or in vitro systems that provided genetic, epigenetic, molecular, or functional insights relevant to keratoconus pathophysiology. Case reports were selectively included when they described rare variants or syndromic associations with clear mechanistic relevance. Exclusion criteria included reports lacking molecular or functional relevance, non–peer-reviewed publications, preprints, and articles published in languages other than English. Data from genome-wide association studies, candidate-gene and family-based linkage analyses, methylation and transcriptomic profiling, and functional or biomechanical assays were extracted and summarized qualitatively. Given the narrative scope of the review, no formal study selection workflow was applied. The extracted information was analyzed qualitatively to provide a coherent overview of the current state of knowledge. Rather than formal data extraction or risk-of-bias assessment, key findings were qualitatively integrated across studies to highlight converging genetic loci, epigenetic mechanisms, molecular pathways, and translational implications. No formal systematic review protocol (e.g., PRISMA guidelines) was applied, consistent with the narrative scope of the manuscript. This narrative approach allowed integration of diverse data types—from genomics to biomechanics—and identification of key knowledge gaps and future research priorities in keratoconus pathogenesis.

## 3. Genetic Variants Identified in Keratoconus

### 3.1. Common Risk Variants (GWAS and Candidate Gene Findings)

Recent genome-wide association studies have significantly expanded our understanding of keratoconus genetics, identifying a number of common variants that confer elevated disease risk. Notably, a 2017 systematic review and meta-analysis of genetic association studies highlighted eight top SNPs across six loci as reproducible risk factors for KC in populations of European descent [9]. These included polymorphisms in or near the genes *FOXO1*, *COL5A1* (in a locus with the retinoid receptor *RXRA*), *FNDC3B*, *IMMP2L*, and the *BANP-ZNF469* region, all initially discovered through GWAS, as well as variants in *COL4A4* identified by earlier candidate-gene studies [10]. *COL5A1* represents one of the most consistently supported loci, with replication across multiple GWAS and case–control studies and biological plausibility based on its established role in collagen fibrillogenesis and corneal structure. Several loci were shared with those influencing central corneal thickness (CCT), a quantitative trait closely related to keratoconus. Indeed, variants in *LOX*, *ZNF469*, *FNDC3B*, *COL5A1*, *FOXO1*, and near *PNPLA2* have been associated with both KC risk and thinner CCT, implicating genes that affect corneal structural integrity [11,12]. FNDC3B has been identified in GWAS of keratoconus and corneal traits, although its functional role in corneal biology remains incompletely characterized. The convergence of KC and CCT loci underscores that genetic factors predisposing to a thinner, biomechanically weaker cornea can increase keratoconus susceptibility.

Specific genes identified by GWAS point to biologically plausible pathways. LOX, for instance, encodes lysyl oxidase, an enzyme critical for collagen cross-linking in the corneal stroma. A significant GWAS hit in the *LOX* gene (e.g., SNP rs2956540) was first linked to KC through a family-based linkage peak on 5q32–33 [13,14]. Subsequent replication in case–control cohorts of European, Chinese, and Iranian patients confirmed the association of a *LOX* variant with keratoconus [15,16,17]. Functionally, *LOX* is responsible for enzymatic cross-linking of collagen and elastin fibers, and reduced *LOX* activity can lead to weaker corneal collagen architecture. Consistent with this, corneal tissue studies show LOX expression and activity are decreased in keratoconic corneas, correlating with disease severity [18]. The association between LOX and keratoconus is supported by replicated findings across multiple populations and is further strengthened by functional evidence demonstrating reduced LOX expression and activity in keratoconic corneas.

Similarly, the *ZNF469* locus has emerged as important from both common-variant and rare-variant perspectives. *ZNF469* (which encodes a large zinc-finger protein) was initially known for its role in the recessive Brittle Cornea Syndrome, but a common SNP (rs9938149) near *ZNF469/BANP* also shows genome-wide significance for keratoconus and CCT. *ZNF469* is thought to regulate extracellular matrix organization (possibly acting as a transcription factor affecting collagen genes), and mutations in *ZNF469* cause extreme corneal thinning in brittle cornea syndrome [9,19]. At the same time, sequencing studies of *ZNF469* in KC cohorts found no strong enrichment of rare, damaging coding variants, suggesting the locus may act primarily via regulatory variation [20]. Evidence for *ZNF469* includes strong human genetic support from syndromic disease (brittle cornea syndrome) and replicated GWAS signals, although enrichment of rare pathogenic variants in sporadic keratoconus remains inconsistent across cohorts.

Other GWAS-implicated loci include *FNDC3B* (a gene of unclear function in the cornea), *IMMP2L* (a mitochondrial inner membrane peptidase gene), and a locus near *FOXO1*. *FOXO1* is especially interesting because it encodes a transcription factor involved in stress response and wound healing; the associated variant might alter corneal cell survival pathways [21,22]. *FOXO1* has emerged from GWAS as a replicated susceptibility locus, with additional support from functional studies implicating FOXO1 in corneal wound healing and stress-response pathways.

Meanwhile, candidate-gene studies (both hypothesis-driven and those guided by linkage intervals) have added additional genes to the KC landscape. *VSX1* (Visual System Homeobox 1) was one of the first candidate genes proposed for keratoconus, based on rare familial cases. *VSX1* encodes a homeodomain transcription factor expressed in the cornea and retina, and some early reports described missense mutations (e.g., R166W, L159M) in *VSX1* in KC patients [23]. However, the role of *VSX1* in keratoconus has proven highly controversial, with many studies yielding conflicting results regarding whether *VSX1* variants are truly pathogenic [24].

Meta-analyses and large screenings have not consistently replicated VSX1 mutations in KC, suggesting that if *VSX1* plays a role, it is likely limited to a small subset or acts in conjunction with other factors. To date, *VSX1* is recognized as a possible but not definitive KC gene—some mutations in *VSX1* may cause atypical corneal dystrophies (like posterior polymorphous corneal dystrophy) that can co-occur with keratoconus, but the majority of sporadic KC cases do not seem to be explained by *VSX1* mutations [25,26,27]. Although early studies proposed *VSX1* as a keratoconus gene, subsequent investigations have yielded inconsistent results, and current evidence suggests that VSX1 is not a major contributor to sporadic keratoconus in most populations.

Beyond *VSX1*, several other candidate genes identified through linkage or association include *TGFBI*, *COL4A3/COL4A4*, *IL1A/IL1B*, *HGF*, and *WNT10A*, among others. Variants in the HGF gene region (encoding hepatocyte growth factor) have been reported in multiple cohorts—for example, a SNP upstream of *HGF* (rs3735520) was associated with keratoconus in studies from the USA, Australia, and Europe [28,29]. HGF is a growth factor known to influence corneal epithelial and stromal biology, and interestingly, it has also been linked to refractive error and corneal thickness traits [30,31]. Associations at the *HGF* locus have been reported in multiple cohorts; however, replication and effect estimates have varied across populations, and overall support is stronger for some GWAS-derived loci than for earlier candidate-gene signals. However, results have varied, and meta-analysis did not find significant pooled association for some earlier reported genes including *HGF*, *IL1A*, and *IL1B* [11], underscoring the need for replication. Meanwhile, genes in the Wnt signaling pathway have gained attention: one study found that polymorphisms in *WNT10A* and *WNT7B* were associated with keratoconus and central corneal thickness, dovetailing with transcriptomic evidence of Wnt pathway dysregulation in KC corneas [32,33,34]. *WNT10A* has been implicated by genetic association studies linking an exonic variant to keratoconus risk through reduced central corneal thickness, with additional pathway-level evidence supporting a role for Wnt signaling dysregulation in keratoconus.

Overall, GWAS and candidate studies in diverse populations (European, Asian, Middle Eastern, etc.) have converged on a set of genes related to ECM structure (collagens *COL5A1*, *COL4A4*), ECM remodeling enzymes (LOX), transcription factors (*FOXO1*, *ZNF469*, *VSX1*), and growth factors/signaling molecules (*HGF*, *WNT10A*) as important players in KC risk. Importantly, the genetic associations summarized in this section are derived predominantly from human population-based studies, including GWAS and large case–control cohorts. It is noteworthy that many KC-associated genes also affect other ocular or connective tissue traits, reinforcing the concept that keratoconus lies at an intersection of pathways governing corneal biomechanics, transparency, and repair [35].

### 3.2. Rare Variants and Overlap with Mendelian Syndromes

In addition to common polygenic risk factors, rarer genetic variants—including those causing Mendelian syndromes—have provided crucial insights into keratoconus pathogenesis. Perhaps the clearest example is Brittle Cornea Syndrome (BCS), a rare recessive connective tissue disorder characterized by extreme corneal thinning and fragility. BCS can be caused by biallelic loss-of-function mutations in *ZNF469* or in *PRDM5*, and patients often present with corneas so thin (200–450 μm or less) that they risk spontaneous rupture [36]. The overlap in corneal phenotype between BCS and keratoconus suggested *ZNF469* as a candidate for the common form of KC. While early screenings for coding mutations in *ZNF469* in sporadic KC were mostly negative [37] some evidence indicates enrichment of rare *ZNF469* variants in some keratoconus cohorts [38].

Beyond these structural genes, a frameshift or missense mutation in *MIR184*, the gene for microRNA-184, causes a familial syndrome of early-onset anterior polar cataract and keratoconus (sometimes called the EDICT syndrome) [39,40]. In these families, a single base substitution in the seed region of *MIR184* (miR-184) segregates with disease, disrupting miR-184’s normal interaction with target mRNAs in the cornea and lens. The mutant miR-184 fails to properly compete with miR-205 for binding sites in genes like *INPPL1* and *ITGB4*, leading to cornea- and lens-specific effects due to miR-184’s high expression in those tissues.

Keratoconus is also observed at higher frequency in several systemic connective tissue disorders. Ehlers–Danlos Syndromes (EDS), a group of heritable collagen disorders, have long been anecdotally linked to keratoconus (patients with joint hypermobility and skin elasticity sometimes have KC). A large sequencing study in 2021 provided strong evidence of a shared genetic etiology between KC and EDS: in a cohort of approximatively 745 KC patients (and 810 controls), researchers resequenced 34 candidate genes and found a significant enrichment of rare variants in *COL5A1*, *COL12A1*, *TNXB*, and *ZNF469*—all genes known to cause various subtypes of EDS [41]. The top single-variant hit was a common variant in *COL12A1* (collagen XII), and gene-based burden tests also pointed to *COL2A1* (collagen II). Notably, *COL5A1* and *COL12A1* mutations underlie classical EDS, *TNXB* mutations cause a form of EDS with tenascin-X deficiency, and *ZNF469* as mentioned is linked to brittle cornea syndrome (sometimes considered an EDS-like condition). The study concluded that genetic variants in EDS genes play a consistent role in KC.

Additional rare variant findings come from family-based research. Linkage analysis in multigenerational KC families has mapped loci on chromosomes 2p24, 3p14–q13, 5q14.3–q21.1, 13q32, 16q22–q23, and 20q12 [42]. While many causative genes in those intervals remained elusive, one notable success was the identification of a segregating mutation in the DOCK9 gene in a large Ecuadorian family [43]. *DOCK9* encodes a guanine nucleotide exchange factor involved in cytoskeletal regulation (also known as *ZRANB1* or Dedicator of Cytokinesis 9). A rare missense mutation (c.2262A > C, p.Gln754His) was found to co-segregate with keratoconus in that family, and functional assays showed it leads to aberrant splicing with exon skipping [44]. The truncated *DOCK9* protein likely loses function in activating the small GTPase Cdc42, which is important for corneal wound healing processes. Although this particular mutation was extremely rare, it provided a clue that cytoskeletal and wound-repair pathways could be relevant to KC—a theme echoed by other findings.

Rare heterozygous mutations in *TGFBI* (which typically cause lattice or granular corneal dystrophies) have occasionally been reported in keratoconus patients too, though it is unclear if these are causal or coincidental [45]. Likewise, keratoconus occurs at higher frequency in Down syndrome (trisomy 21) [46], and in Leber congenital amaurosis (particularly in those with *CRB1* mutations), hinting at genetic pathways (perhaps collagen, extracellular matrix, or developmental pathways) that intersect with KC [47]. Table 1 summarizes the principal genetic findings in keratoconus, including variant type, primary evidence source, associated biological pathways, and replication status.

Collectively, these Mendelian and familial findings underscore that disruption of corneal extracellular matrix organization, collagen cross-linking, and cytoskeletal integrity represents a convergent theme in keratoconus pathogenesis. The evidence discussed in this section is largely based on human familial, syndromic, and sequencing studies, with functional insights in some cases supported by in vitro or animal experiments. Insights from such monogenic disorders are now being leveraged in large-scale exome and genome sequencing efforts to identify ultra-rare, high-impact variants in broader KC cohorts, aiming to bridge the gap between Mendelian mechanisms and complex polygenic risk.

## 4. Epigenetic and Transcriptomic Factors

While DNA sequence variants set the stage for keratoconus susceptibility, epigenetic modifications and gene expression changes are emerging as key contributors to the disease’s development and progression. Epigenetic and transcriptomic findings in keratoconus arise from a combination of studies in human corneal tissue and tear fluid, as well as from in vitro and animal models. Epigenetics refers to heritable changes in gene function that do not involve alterations of the DNA sequence itself—principally DNA methylation, histone modifications, and non-coding RNA regulation [48]. Importantly, most epigenetic and transcriptomic studies in keratoconus are cross-sectional and performed on already diseased corneal tissue, often obtained at advanced stages. As a result, observed DNA methylation and non-coding RNA changes should primarily be interpreted as associative, and may reflect secondary or compensatory responses to disease-related stress, inflammation, or tissue remodeling rather than primary causal events.

These mechanisms can dynamically respond to environmental and developmental cues, providing a link between external factors (like inflammation or mechanical stress) and the genomic machinery of the cell. In the cornea, epigenetic changes have been increasingly studied to explain how gene–environment interactions might lead to the thinning and cone formation of KC. Here we review the current knowledge on epigenetic and transcriptomic alterations in keratoconus, including DNA methylation patterns, histone modification findings, and the role of non-coding RNAs (microRNAs and long non-coding RNAs) in corneal tissue.

### 4.1. DNA Methylation and Histone Modification Changes

DNA methylation, typically the addition of methyl groups to cytosines in CpG dinucleotides, is a major regulator of gene expression. Two cornea-specific studies in recent years have examined the DNA methylation landscape in keratoconus corneas, revealing differential methylation that correlates with known genetic risk loci and dysregulated pathways. Notably, the first genome-wide methylation analysis of keratoconus tissue was published in 2019 by Kabza et al. [8], who performed reduced-representation bisulfite sequencing on KC vs. control corneas. They identified multiple keratoconus-specific differentially methylated regions (DMRs)—clusters of CpG sites with altered methylation in KC—many of which overlapped previously identified KC linkage loci on chromosomes 3p, 5q, 13q, 15q, 20p, etc. In fact, several chromosomal arms that had shown linkage to KC (e.g., 2q, 4q, 14q, 17q) harbored significant DMRs in the study.

This overlap suggests that areas of the genome implicated by inheritance also undergo epigenetic modulation in disease states. Furthermore, by re-analyzing corneal RNA-seq data, the authors found that a number of genes with altered expression in KC (12 downregulated and 6 upregulated) were located near these DMRs. Particularly intriguing were DMRs found in the vicinity of *WNT3* and *WNT5A*, two genes encoding Wnt signaling ligands. Given Wnt pathways’ role in cell differentiation and fibrosis, such epigenetic alteration provides a mechanistic clue for the Wnt pathway activation seen in KC pathology. The study concluded that keratoconus corneas exhibit distinct patterns of DNA hypo- and hypermethylation that likely influence gene expression and can even “explain” some of the genetic linkage findings (i.e., the methylation changes may tag regulatory regions in those linked loci).

Another recent multi-omics analysis reported methylation changes in *LOX*, *SMAD3*, *KLF5*, *HOXB1*, and *BANP* among others in keratoconus tissue [12]. Interestingly, this analysis integrated an epigenetic analysis and found that keratoconus-associated SNPs were enriched for effects on DNA methylation: for example, risk alleles at one locus significantly influenced methylation of the transcription factor gene KLF4 in corneal-relevant tissue. *KLF4* is a critical transcription factor for corneal epithelial homeostasis, and differential methylation of *KLF4* could affect corneal epithelial integrity in KC.

Histone modifications (such as acetylation or methylation of histone tails) have been less directly studied in keratoconus, but they are another likely factor. Epigenetic reviews note that altered histone acetylation can change chromatin accessibility and thus gene expression in corneal diseases [49]. One example in the literature is the gene *DPF3*, which encodes a histone acetyl-lysine reader protein. A locus associated with keratoconus in one study was near *DPF3*, hinting that variations in chromatin reader proteins could play a role [50]. Although specific histone marks have not yet been mapped genome-wide in KC corneas, experimental therapies targeting histone deacetylases (HDACs) are being considered in corneal scarring and might eventually be relevant to keratoconus if aberrant chromatin marks are identified [51,52]. However, it should be emphasized that current DNA methylation data in keratoconus are largely derived from cross-sectional analyses of affected corneal tissue and do not establish temporal or causal relationships. Tissue heterogeneity, differences in disease severity, and the influence of environmental factors such as mechanical stress or inflammation may contribute to the observed methylation patterns. Longitudinal studies and functional experiments manipulating methylation at specific loci will be required to determine whether these epigenetic alterations play a primary pathogenic role or arise secondary to disease progression.

### 4.2. Non-Coding RNAs (miRNAs, lncRNAs) and Gene Expression Profiles

Non-coding RNAs have emerged as important regulators of gene expression in keratoconus, especially microRNAs (miRNAs). The cornea normally expresses a rich repertoire of miRNAs (nearly 300 different ones, with about 18 being cornea-specific) [51]. MiR-184 is highly expressed in the corneal epithelium (particularly in the central basal layers), and as discussed, a *MIR184* mutation can cause familial KC with cataract [39,40,52]. Even in typical KC cases without *MIR184* mutations, there is growing evidence that global miRNA expression is perturbed in keratoconus. A recent review summarized studies on miRNA profiling in KC and found that overall, 29 microRNAs were upregulated and 11 were downregulated in keratoconic tissues compared to normals [51]. Among the consistently upregulated were miR-143-3p, miR-182-5p, and miR-92a-3p, while many downregulated miRNAs were ones associated with maintaining cell–cell junctions, cell cycle, and cytoskeletal organization (processes important for corneal structure). For example, miR-29 family and miR-133 (implicated in fibrosis and muscle differentiation, respectively) have been noted as altered in some KC studies [7].

In less advanced KC, changes in a small set of miRNAs (miR-151a-3p, miR-185-5p, miR-194-5p, miR-195-5p) were observed specifically in the cone epithelium, whereas in advanced KC, a broader array of miRNAs (including miR-138-5p, miR-146b-5p, miR-181a-2-3p, miR-28-5p) showed dysregulation [51]. This suggests the miRNA expression profile evolves as the disease progresses, possibly reflecting the ongoing stress and remodeling of the cornea. The functional consequences of these miRNA changes are under active investigation: many of the upregulated miRNAs (like miR-143 and miR-182) are known to target mRNAs involved in ECM production and apoptosis, potentially promoting matrix degradation or keratocyte loss when overexpressed. Conversely, downregulation of miRNAs that normally restrain inflammation (e.g., miR-146b) could lead to unchecked inflammatory mediator expression. One hypothesis posits that keratoconus may be driven by a “dynamic miRNA signature” in the corneal epithelium that shifts in response to chronic injury (rubbing, UV, etc.), leading to aberrant wound healing and thinning.

Efforts are underway to identify specific miRNA biomarkers in easily accessible samples (like tears or even blood). For instance, distinct miRNA profiles in the tear fluid of KC patients have been proposed as non-invasive biomarkers for early detection [53].

Long non-coding RNAs (lncRNAs), which are mRNA-length transcripts that do not code proteins, have also been examined. A 2020 RNA-sequencing study compared the corneal transcriptome (both mRNAs and lncRNAs) of KC versus healthy corneas and found many differentially expressed lncRNAs that correlated with coding-gene changes [32]. Furthermore, a dedicated resource, KTCNlncDB, has been developed to catalog lncRNAs identified in KC corneal samples [54]. While the functions of these lncRNAs remain largely uncharacterized, it is plausible that some regulate the expression of structural genes or matrix-modifying enzymes in the cornea. For instance, lncRNAs may act as competing endogenous RNAs (ceRNAs) that sequester miRNAs, thereby indirectly modulating the translation of collagen or protease transcripts. Recent bioinformatic analyses have proposed lncRNA–miRNA–mRNA regulatory networks in keratoconus, implicating pathways such as TGF-β and Hippo signaling [55,56]. As data accumulate, certain lncRNAs could become novel biomarkers or therapeutic targets.

In terms of broad transcriptomic changes, beyond specific non-coding RNAs, keratoconus corneas exhibit a consistent pattern of mRNA expression changes reflecting the disease’s hallmark processes. Several gene expression studies (using microarrays and RNA-seq) have shown downregulation of collagens and keratan sulfate proteoglycans (which make up the corneal stroma), along with upregulation of proteinases and inflammatory cytokines 547 genes associated with immune and inflammatory responses (leukocyte adhesion, cytokine receptors, etc.) were identified in a recent transcriptomic study [57,58].

**Figure 1:**
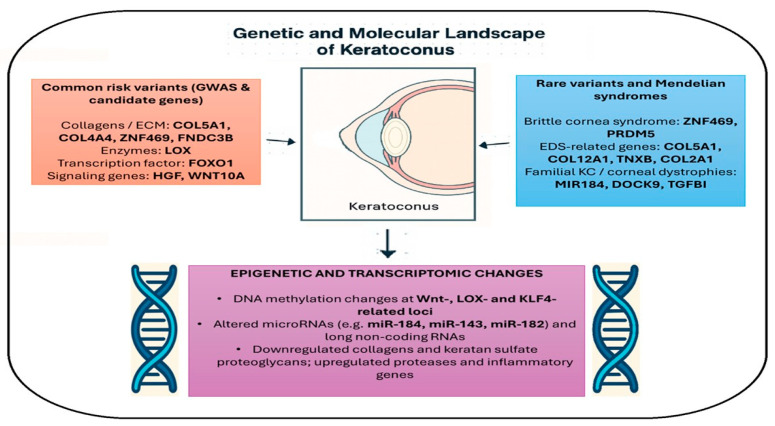
Genetic and epigenetic architecture of keratoconus. Schematic representation of the main biological pathways implicated in keratoconus. Common risk variants identified by GWAS and candidate-gene studies cluster in extracellular matrix and signaling genes (*COL5A1*, *COL4A4*, *ZNF469*, *FNDC3B*, *LOX*, *FOXO1*, *HGF*, *WNT10A*), many of which are shared with loci influencing reduced central corneal thickness. Rare variants overlap with Mendelian connective-tissue and ocular disorders, including brittle cornea syndrome (*ZNF469*, *PRDM5*), Ehlers–Danlos–related genes (*COL5A1*, *COL12A1*, *TNXB*, *COL2A1*), and familial keratoconus or corneal dystrophies (*MIR184*, *DOCK9*, *TGFBI*). Epigenetic and transcriptomic alterations—such as DNA methylation changes at Wnt-, LOX- and KLF4-related loci, dysregulated microRNAs (e.g., miR-184, miR-143, miR-182) and long non-coding RNAs, with downregulated collagens/proteoglycans and upregulated proteases and inflammatory genes—further contribute to corneal weakening and disease progression.

Epigenetic deregulation and non-coding RNAs orchestrate the mis-expression of genes that govern corneal strength and homeostasis. DNA methylation studies directly tie environmental or developmental influences to known genetic hotspots in KC (e.g., Wnt pathway genes). However, most studies reporting altered microRNA and long non-coding RNA expression in keratoconus are based on cross-sectional analyses of corneal epithelium, stroma, or tear fluid from affected eyes. While these findings demonstrate reproducible associations with disease state, they do not establish causality, as changes in non-coding RNA expression may represent downstream effects of chronic injury, oxidative stress, or aberrant wound healing. Although functional studies in experimental models suggest that certain microRNAs can modulate extracellular matrix remodeling and apoptosis, direct causal evidence in human keratoconus remains limited. MicroRNA and lncRNA findings offer an additional post-transcriptional layer by which corneal cells might fail to produce adequate matrix or might over-produce degrading enzymes. These molecular aberrations, in combination with the hardwired genetic variants described earlier, set the stage for the phenotype of keratoconus. Figure 1 summarizes the main genetic factors associated with keratoconus and their downstream epigenetic and transcriptomic changes.

## 5. Gene–Environment Interactions

Keratoconus has long been thought to result from an interplay between genetic susceptibility and environmental/behavioral triggers. Eye rubbing is perhaps the most well-established environmental risk factor—many KC patients have a history of vigorous or chronic eye rubbing, and it has been postulated that mechanical trauma to the cornea can induce micro-damage that accelerates ectasia [59]. Atopy is another recognized risk factor, potentially because atopic individuals often suffer from allergic ocular itching that leads to more rubbing, as well as from baseline elevated inflammatory mediators [60]. Recent epidemiological studies confirm that both factors independently increase keratoconus risk, and importantly, they may act synergistically. In a 2022 case–control study of 330 KC patients and 330 controls in China, approximatively 69% of KC patients had a significant history of eye rubbing and approximatively 16% had atopy, both markedly higher than in controls [61].

Gene–environment interaction studies are now beginning to pinpoint specific genetic variants that modify the impact of environmental factors like eye rubbing. A notable recent example involves the calpastatin gene (*CAST*), which encodes an inhibitor of calpains (calcium-dependent proteases). The *CAST* locus on 5q was linked to KC in two independent linkage studies, and *CAST* variants showed association in follow-up family-based and case–control analyses [62,63]. Calpains are thought to contribute to keratocyte apoptosis and tissue remodeling in the cornea. A 2024 study investigated whether *CAST* gene polymorphisms interact with eye rubbing behavior to influence KC risk. Using a case-only design with 930 KC patients (comparing those who rub vs. those who do not rub), they found three SNPs in *CAST* (rs26515, rs27991, rs9314177) with significant gene–environment interaction effects [64]. Intriguingly, the minor alleles of these SNPs had negative interactions with eye rubbing—meaning that among eye rubbers, carrying the risk allele was less harmful than expected, suggesting a complex interplay where certain *CAST* genotypes might mitigate rubbing damage or vice versa. Logistic regression confirmed that individuals homozygous or heterozygous for the minor alleles of those SNPs exhibited a different risk profile when rubbing was present. A multi-locus analysis further supported SNP–SNP–environment interactions between the three *CAST* variants and rubbing. While the biology needs elucidation, one hypothesis is that some *CAST* variants might lead to higher calpastatin levels or activity, thereby protecting the cornea from protease-mediated damage during rubbing episodes. This is among the first demonstrations of a gene–environment interaction at the molecular level in keratoconus, highlighting that the effect of environmental stresses can depend on one’s genotype.

Beyond eye rubbing and atopy, other environmental and lifestyle factors under investigation include UV exposure [65], contact lens wear [66], and other systemic factors have been proposed such as hormonal factors, nutritional and obesity [67,68,69].

Crucially, gene–environment interplay might also operate through epigenetics. Environmental triggers like chronic rubbing or allergy-associated inflammation can alter epigenetic marks in corneal cells—for example, repetitive mechanical stress could lead to local DNA methylation changes or miRNA expression shifts [51,70]. Over time, these acquired epigenetic modifications could lock in a pathological gene expression pattern that perpetuates keratoconus even if the inciting behavior stops. This concept aligns with the earlier discussion of how environmental factors might cause “dynamic” changes in the corneal epithelium’s gene regulatory network. Environmental and behavioral risk factors implicated in keratoconus, such as chronic eye rubbing, atopy, and UV exposure, can be mechanistically linked to several of the molecular and epigenetic pathways discussed above. Mechanical stress induced by eye rubbing has been shown to cause epithelial injury, keratocyte apoptosis, and activation of inflammatory signaling pathways, with increased expression of cytokines and matrix-degrading enzymes that contribute to extracellular matrix remodeling, similarly, atopy-associated ocular surface inflammation is characterized by elevated pro-inflammatory mediators, which are known to influence oxidative stress responses and gene regulatory networks in corneal cells [61]. Oxidative stress, a well-established feature of keratoconus and a consequence of UV exposure [65]. Reactive oxygen species mayalter epigenetic marks and microRNA profiles, and such mechanisms may contribute to the epigenetic and transcriptomic alterations observed in keratoconic corneas. While direct causal links between specific environmental exposures and defined epigenetic changes in keratoconus have not been conclusively demonstrated, the convergence of mechanical stress, inflammation, oxidative injury, and dysregulated gene expression supports a biologically coherent model in which environmental triggers interact with genetic and epigenetic susceptibility to drive disease progression.

Substantial evidence confirms that environmental factors like eye rubbing and atopy significantly heighten the risk of keratoconus, especially on the background of genetic susceptibility. Public health and clinical advice for KC patients (and high-risk individuals) consistently includes avoiding eye rubbing—an intervention that could theoretically slow disease progression. Furthermore, the emerging gene–environment interaction findings (e.g., *CAST* gene) raise the prospect of precision medicine approaches: for example, identifying individuals who’s genetic makes them particularly vulnerable to environmental insults, and counseling them on preventive measures. The combination of controlling environmental triggers and understanding a patient’s genetic risk profile may prove most effective in managing early keratoconus.

## 6. Molecular Pathways and Functional Mechanisms

The genetic and epigenetic factors described above ultimately manifest in keratoconus as changes in cellular and biochemical pathways within the cornea. Key among these are processes related to the ECM remodeling, collagen crosslinking, oxidative stress, and inflammation/wound healing. The molecular pathways described below integrate evidence from human corneal samples, animal models, and in vitro systems. Although these complementary approaches support convergent biological themes, differences in experimental context and disease representation limit direct causal inference and clinical generalization. Understanding these pathways is critical, as they form the bridge from gene-level changes to the macroscopic clinical findings (thinning, bulging, scarring).

### 6.1. Extracellular Matrix Remodeling and Collagen Crosslinking

A unifying theme in keratoconus is a weakening of the corneal stromal scaffold, which is principally composed of collagen fibrils (types I and V collagen) and interwoven proteoglycans (keratan sulfate proteoglycans like lumican, keratocan, decorin) [71]. Genetic studies have repeatedly pointed to disruptions in matrix components or their regulators [10,11,12,35]. Another aspect of ECM remodeling in KC is the overactivity of proteases and underactivity of their inhibitors. Beyond *LOX*, studies have shown altered expression or function of proteolytic enzymes such as calpains, cathepsins, and matrix metalloproteinases (MMPs) [72]. For example, calpain small subunit 1 was found at higher levels in KC corneal epithelium [73]. Similarly, cathepsin V/L2 was reported to be elevated in KC corneas, whereas natural inhibitors were reduced [74]. Matrix metalloproteinases (especially MMP-9 and MMP-13) are often upregulated in keratoconus; these enzymes cleave collagens and basement membrane components. Tellingly, tear fluid of KC patients contains elevated MMP-9 levels, and treatment with cross-linking or contact lenses can reduce those levels over time, indicating MMPs are dynamically involved in corneal remodeling [75,76].

### 6.2. Oxidative Stress, Inflammation, and Wound-Repair Pathways

Another critical pathogenic mechanism in keratoconus is oxidative stress—essentially, an imbalance between the production of reactive oxygen (ROS) and the cornea’s antioxidant defenses [77,78]. The cornea is continually exposed to UV light and high oxygen tension, which can generate ROS. Under normal circumstances, corneal cells express abundant antioxidants. In keratoconus, multiple lines of evidence show that these protective systems are compromised [79,80]. The genetic association with *SOD1* further highlights the importance of oxidative pathways. Some studies reported rare *SOD1* variants in KC patients or altered SOD1 activity, though these findings are not yet conclusive [81,82,83]. Given the documented oxidative-stress burden in keratoconus, treatments aimed at enhancing corneal antioxidant defenses have been proposed and explored in experimental models, although direct clinical trials in KC remain scarce [84,85,86]. Hand-in-hand with oxidative stress is inflammation, though keratoconus is traditionally labeled “non-inflammatory” because it lacks obvious immune cell infiltration. We now know that subclinical inflammation does exist in KC corneas. Tear fluid analyses have shown elevated levels of inflammatory cytokines such as IL-6, TNF-α, IL-1β, and matrix metalloproteinases in KC patients [87,88,89]. Immunohistochemical and ocular surface analyses in keratoconus have demonstrated up-regulation of IL-6, IL-17A and TNF-α in the epithelium, stroma or tear film, along with increased immune-cell activation (including NK cells/γδT cells), supporting a role for low-grade inflammation in the disease process [90,91].

**Figure 2:**
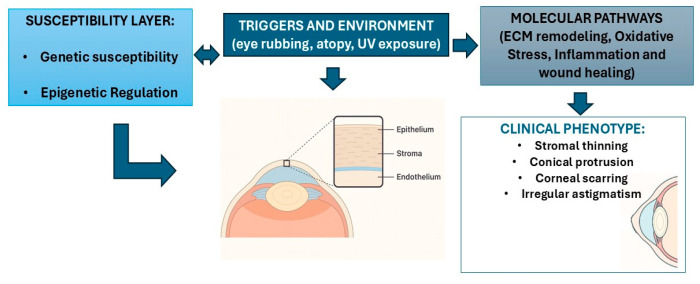
Integrated model of keratoconus pathogenesis linking genetic/epigenetic susceptibility, environmental triggers, and corneal biomechanical failure.

Wound-healing pathways are also perturbed in keratoconus. The cornea normally responds to injury by activating keratocytes to become myofibroblasts and secrete new matrix. In keratoconus, histology and molecular studies document keratocyte apoptosis (including in Bowman’s layer) and defective wound-repair signaling. For example, *VSX1*—studied in KC—has been shown to be expressed in keratocyte/myofibroblast wound-healing models [92], while *DOCK9* (via Cdc42) has been implicated in corneal wound closure signaling [44]. Dysfunction in these genes may impair micro-wound healing, compounding the mechanical and structural damage characteristic of KC. In summary, keratoconus represents a maladaptive corneal repair process in which the extracellular matrix is continuously degraded but insufficiently rebuilt. Oxidative stress overwhelms antioxidant defenses, and low-grade inflammation persists, especially under mechanical stress. Genetic predisposition provides structural fragility, environmental factors such as eye rubbing act as triggers, and epigenetic and non-coding RNA mechanisms amplify the response. The outcome is progressive stromal thinning and biomechanical failure, forming the characteristic cone. In Figure 2, we illustrate the main factors involved in the pathogenesis of keratoconus, genetic/epigenetic susceptibility, environmental triggers and biomechanical failure of the cornea.

## 7. Clinical Translation and Future Directions

The advances in genetic and epigenetic research on keratoconus are beginning to be translated into clinical applications aimed at early diagnosis, risk stratification, and personalized treatment. While the primary therapy for progressive keratoconus today remains corneal collagen cross-linking (plus visual rehabilitation with specialty contact lenses or transplantation in late stages), the hope is that molecular insights will enable intervention before significant vision loss occurs and perhaps even prevent the disease in high-risk individuals.

### 7.1. Early Diagnosis and Risk Prediction (Polygenic Scores and Biomarkers)

One of the most immediate benefits of understanding keratoconus genetics is the potential to identify individuals at high risk before clinical signs fully develop. Keratoconus often starts in adolescence and can progress rapidly; therefore, knowing who is genetically predisposed could prompt earlier corneal imaging and prophylactic measures (like avoiding eye rubbing or earlier cross-linking). Traditional family history has limited utility, so genetic risk scores have been proposed. In 2025, He et al. [93] published the development of a comprehensive polygenic risk score (PRS) for keratoconus leveraging GWAS data and related corneal traits. By integrating multiple associated variants (and even using multi-trait analysis with corneal curvature and thickness data), they created PRS models that were tested in large biobank cohorts. The best model achieved an odds ratio of approximately 2.3 per standard deviation of PRS in an Estonian cohort, and when combined with age and sex the PRS-based model had an AUC (area under the curve) of 0.72 for predicting KC. In the UK Biobank dataset, adding the PRS to clinical corneal measurements significantly improved prediction AUC from 0.84 to 0.88 (which is a substantial gain). This proof-of-concept shows that a polygenic risk model can stratify KC risk with reasonable accuracy. Such a tool could be used, for example, in screening refractive surgery candidates: a high PRS might contraindicate LASIK, as those patients are at higher risk for postoperative ectasia. It could also be used in the general population to flag young people for early topography screening, akin to how genetic scores are considered for other complex diseases. However, current models still face important limitations: their predictive power remains insufficient for standalone clinical use; most training data derive from European ancestry cohorts, limiting generalizability; and PRS performance depends heavily on accurate phenotyping and harmonized corneal trait measurements. Larger, multi-ethnic datasets and integration with clinical or imaging biomarkers will be required before PRS-based screening can be reliably implemented in ophthalmic practice. An important limitation of the current genetic literature on keratoconus is the limited ancestral diversity of available cohorts. Most genome-wide association studies and sequencing efforts to date have been conducted in populations of European or East Asian descent, with relatively sparse representation of individuals from African, Latin American, Middle Eastern, or South Asian backgrounds. As a consequence, the transferability of identified risk loci and, in particular, the clinical performance of polygenic risk scores may be reduced in underrepresented populations due to differences in allele frequencies, linkage disequilibrium structure, and gene–environment interactions. This limitation has important implications for the equitable clinical implementation of genetic risk prediction tools. Future studies will require large, well-phenotyped, multi-ethnic cohorts and ancestry-aware modeling approaches to improve the generalizability, accuracy, and fairness of keratoconus genetic risk stratification.

Beyond DNA-based prediction, biomarker discovery is in progress for keratoconus. Given that the disease affects accessible tissues (cornea, tear fluid), there is interest in developing assays that detect molecular signs of KC even before shape changes are evident on topography. For instance, analysis of tear film components in keratoconus has revealed candidate biomarkers: elevated levels of certain cytokines (IL-6, TNF-α), MMPs, and specific microRNAs. As mentioned, a panel of differentially expressed miRNAs in tears has been proposed as an early diagnostic signature [53]. Oxidative-stress biomarkers (such as lipid peroxidation markers and antioxidant enzyme activities) are elevated in the tear fluid and corneal tissue of keratoconus patients, suggesting ongoing redox injury. Minimally invasive sampling methods—such as tear collection or impression cytology of superficial corneal cells—permit molecular profiling (e.g., non-coding RNA or DNA methylation) and hold promise as adjunct diagnostics [94].

In terms of personalized risk assessment, the future likely holds integrated models that combine genetic data, demographic factors, biochemical markers, and corneal imaging metrics. Additionally, for patients who already have KC, genetics can inform management—e.g., identifying those with underlying EDS gene mutations who might also have systemic issues like joint hypermobility, guiding referrals to rheumatology or avoidance of certain surgeries. The presence of a *TNXB* or *COL5A1* mutation (related to EDS) in a keratoconus patient might prompt screening for mitral valve prolapse or vessel fragility, for instance. This holistic, personalized approach is gradually coming into view as research ties specific molecular profiles to prognosis.

### 7.2. Toward Personalized Therapies

Current keratoconus treatments (e.g., contact lenses, corneal cross-linking, intrastromal rings, keratoplasty) do not directly correct the underlying molecular defects. Given accumulating data on dysregulated microRNAs and gene expression in KC corneas, RNA-based therapies are being discussed as a future avenue. Profiling studies show altered expression of multiple miRNAs (including miR-182-5p and others) in KC tissue, supporting the idea that post-transcriptional regulation contributes to extracellular matrix remodeling and oxidative stress responses [51].

In parallel, there is growing proof-of-concept from other ocular diseases that synthetic oligonucleotides can be delivered safely to the eye: antisense oligonucleotides and siRNA/shRNA constructs have been used experimentally to suppress pro-angiogenic or pro-fibrotic targets (e.g., IRS-1, TGF-β1/TGFβR2/CTGF, MMP-9) and reduce corneal neovascularization or scarring in animal and in vitro models, and efficient delivery of antisense oligos to the cornea via topical or minimally invasive routes has been demonstrated [95,96,97].

Extrapolating from these data, one can plausibly envision KC-directed approaches—such as siRNA or antisense constructs targeting matrix-degrading enzymes, or oligonucleotides enhancing protective factors like *LOX* or antioxidant pathways—but no RNA-based therapy has yet been tested clinically in keratoconus, and target selection, delivery, off-target effects, and long-term safety all remain major challenges.

Another futuristic approach is gene editing. With CRISPR-Cas technology rapidly advancing in ophthalmology with The first in vivo CRISPR trial (EDIT-101) for CEP290-associated Leber congenital amaurosis 10 initiated in 2020 [98] demonstrating the feasibility of direct gene editing in the human eye. In the cornea, ex vivo studies have shown that CRISPR/Cas9 can precisely correct pathogenic *TGFBI* mutations in patient-derived keratocytes, supporting the concept of mutation-specific gene editing for monogenic corneal dystrophies [99].

These advances make it tempting to speculate about gene editing approaches for keratoconus—such as correcting highly penetrant rare variants or using CRISPR interference/activation systems (CRISPRi/CRISPRa) to downregulate matrix-degrading enzymes or enhance protective factors (e.g., ECM or antioxidant genes) in corneal cells. However, no CRISPR- or gene-editing–based therapy has yet been tested for keratoconus in vivo, and several hurdles remain substantial: KC is predominantly polygenic rather than driven by a single causal mutation; safe, efficient, and layer-specific delivery of editors to corneal epithelium and stroma is not solved; and off-target, on-target-but-undesired edits and long-term safety are critical concerns. The cornea’s accessibility and compartmentalization make it an attractive future target, but at present gene editing in KC should be considered a theoretical, longer-term strategy extrapolated from work in monogenic corneal dystrophies and inherited retinal diseases, rather than an imminent clinical option. Other emerging pharmacologic strategies includes approaches aim to modulate corneal biomechanics or its pathogenic microenvironment rather than replace tissue. One of the most advanced is *LOX*-targeted pharmacologic cross-linking: copper-based eye drops (e.g., IVMED-80) are designed to enhance lysyl oxidase activity and physiologic collagen cross-linking [100]. Preclinical work and an early phase 1/2a trial in keratoconus suggest increased corneal stiffness and modest Kmax flattening, but larger controlled studies are needed.

Antioxidant therapies are supported mainly by experimental and non-KC data: N-acetylcysteine (NAC)–based formulations can reduce oxidative damage and promote corneal healing in animal models and ocular surface disease, yet there are currently no robust clinical trials showing that topical antioxidants slow keratoconus progression [101,102]. Anti-inflammatory treatment (topical steroids, cyclosporine, anti-allergic drops) is already used in KC patients to treat ocular allergy in order to reduce inflammation and eye rubbing, but this is supportive care; long-term disease-modifying effects on KC itself remain unproven.

On the cell therapy side, Stromal cell therapy for keratoconus has moved from concept to early clinical testing. Preclinical studies using corneal stromal stem cells (CSSCs) and mesenchymal stem cells (MSCs) show that these cells can home to the corneal stroma, secrete anti-inflammatory and anti-fibrotic factors, differentiate into keratocyte-like cells, and deposit organized extracellular matrix, partially restoring transparency and stiffness in animal models [103,104,105]. A phase I clinical trial by Alió del Barrio et al. implanted autologous adipose-derived adult stem cells into the corneal stroma of patients with advanced keratoconus and reported the procedure to be feasible and safe, with signals of increased stromal reflectivity/new collagen and mild improvement or stabilization of visual and tomographic parameters over 1–3 years [106,107]. Ongoing and planned studies (e.g., intrastromal ADSC implantation with or without decellularized stromal scaffolds) further support the translational potential of this strategy [108]. These approaches do not correct the underlying polygenic susceptibility but may functionally compensate for dysfunctional keratocytes by repopulating the stroma with matrix-producing, immunomodulatory cells; long-term efficacy, durability of engraftment, and standardization of cell sources and delivery remain key open questions. Despite these promising translational directions, several important challenges must be addressed before genetic and molecular tools can be implemented in routine clinical practice. Current polygenic risk score models for keratoconus have been developed primarily in populations of European ancestry, limiting their generalizability and underscoring the need for large, ethnically diverse cohorts to ensure equitable clinical applicability. Similarly, while tear-based biomarkers show promise, most candidates have been identified in cross-sectional studies and require prospective validation, standardization of sampling methods, and demonstration of clinical utility in early or subclinical disease. Gene- and RNA-based therapeutic strategies face additional hurdles, including safe and efficient delivery to corneal tissues, durability of effect, off-target risks, and long-term safety. Furthermore, the predominantly polygenic nature of keratoconus complicates the application of gene-editing approaches that are more suited to monogenic disorders. Ethical and regulatory considerations—such as genetic data privacy, informed consent for predictive testing, and appropriate patient selection—will also be critical as molecular diagnostics and therapies advance. Addressing these challenges through rigorous validation, inclusive study design, and careful regulatory oversight will be essential to translate molecular insights into safe and effective clinical interventions.

The landscape of keratoconus therapy is expanding beyond mechanical stabilization toward biologically informed and personalized interventions. Advances in genomics, RNA therapeutics, gene editing, pharmacologic modulation, and cell-based regeneration collectively point to a future in which treatment targets the molecular root of disease rather than its structural consequences. While most of these strategies remain in preclinical or early clinical phases, the cornea’s accessibility and the growing understanding of KC pathophysiology make it a uniquely tractable model for precision ophthalmology. Continued integration of genetic, molecular, and biomechanical data will be key to transforming keratoconus management from reactive to preventive and restorative care.

## 8. Conclusions

Keratoconus represents a paradigmatic example of a complex, multifactorial disorder in which genetic predisposition, epigenetic regulation, and environmental influences converge to produce a progressive biomechanical failure of the cornea. Over the past two decades, genome-wide and candidate-gene studies have firmly established the contribution of variants in collagen- and extracellular matrix–related genes, redox regulators, and signaling molecules. Complementary epigenetic and transcriptomic research has revealed disease-associated changes in DNA methylation and non-coding RNA expression that help explain how environmental stresses—such as eye rubbing, atopy, or oxidative injury—interact with genetic susceptibility.

These molecular insights are beginning to reshape the clinical landscape. Polygenic risk scores and tear- or cell-based molecular assays may soon complement topography for early diagnosis and personalized risk prediction. At the same time, translational work in RNA therapeutics, gene editing, and stromal stem cell therapy opens the prospect of biologically targeted and regenerative interventions that address the underlying pathogenic mechanisms rather than the late mechanical consequences. However, the successful clinical translation of these advances will depend on addressing key limitations, including the need for diverse patient cohorts, prospective validation of molecular biomarkers, and careful evaluation of safety, ethical, and regulatory considerations associated with genetic and RNA-based interventions. A major limitation of the current evidence base is the heterogeneity of study designs, populations, disease stages, and experimental models, with many mechanistic insights derived from in vitro or animal studies rather than longitudinal human cohorts. As a result, several proposed pathways and therapeutic targets remain incompletely validated in clinical keratoconus.

Future research should aim to integrate multi-omic, biomechanical, and clinical datasets to refine risk models, validate molecular biomarkers, and guide tailored interventions. By combining preventive strategies with precision molecular therapies, the management of keratoconus could evolve from halting progression to restoring corneal integrity and function.

## Figures and Tables

**Table 1:** Summary of key genetic loci implicated in keratoconus, including variant type, primary evidence source, associated molecular pathways, and replication status across studies.

Gene/Locus	Variant Type	Primary Evidence Type	Associated Pathway(s)	Replication Notes	Key References
*LOX*	Common variants	GWAS, candidate gene, functional studies	Collagen cross-linking, ECM stability	Replicated across multiple populations; reduced expression/activity reported in KC corneas	[13,14,15,16,17,18,25]
*ZNF469*	Common variants; rare variants (syndromic)	GWAS; sequencing in brittle cornea syndrome	ECM organization, collagen regulation	GWAS association replicated; rare variant enrichment inconsistent in sporadic KC	[9,19,20,36,37,38]
*COL5A1*	Common variants	GWAS, case–control studies	Collagen fibrillogenesis, ECM structure	Consistently associated with KC and central corneal thickness	[9,10,11,12,41]
*HGF*	Common variants	Candidate gene studies; GWAS	Growth factor signaling, wound healing	Associations reported in multiple cohorts; replication variable across populations	[11,28,29,30,31]
*VSX1*	Rare variants	Candidate gene, family studies	Transcriptional regulation, wound response	Findings inconsistent; not a major contributor in most sporadic KC cases	[23,24,25,26,27]
*FOXO1*	Common variants	GWAS	Stress response, wound healing	Replicated GWAS signal; functional relevance supported by experimental studies	[9,21,22]
*WNT10A*	Common variants	GWAS, corneal trait association studies	Wnt signaling, corneal development	Implicated through association with corneal thickness; KC-specific replication limited	[32,33,34]
*FNDC3B*	Common variants	GWAS	Cell adhesion, corneal traits (proposed)	Identified in GWAS; functional role in cornea remains unclear	[9,11,12]

## Data Availability

No new data were created or analyzed in this study.

## Abbreviations

BCS	brittle cornea syndrome;
CAST	calpastatin;
CCT	central corneal thickness;
CSSCs	corneal stromal stem cells;
ECM	extracellular matrix;
EDS	Ehlers–Danlos syndromes;
GWAS	genome-wide association studies;
KC	keratoconus;
lncRNAs	long non-coding RNAs;
MMPs	matrix metalloproteinases;
MSCs	mesenchymal stem cells;
PRS	polygenic risk score;
ROS	reactive oxygen species;
SNPs	single-nucleotide polymorphisms.
